# How Integration of a Brain-Machine Interface and Obstacle Detection System Can Improve Wheelchair Control via Movement Imagery

**DOI:** 10.3390/s24030918

**Published:** 2024-01-31

**Authors:** Tomasz Kocejko, Nikodem Matuszkiewicz, Piotr Durawa, Aleksander Madajczak, Jakub Kwiatkowski

**Affiliations:** Department of Biomedical Engineering, Faculty of Electronics, Telecommunications and Informatics, Gdansk University of Technology, Narutowicza 11/12, 80-233 Gdansk, Poland; s175801@student.pg.edu.pl (N.M.); s176300@student.pg.edu.pl (P.D.); s175867@student.pg.edu.pl (A.M.); s192237@student.pg.edu.pl (J.K.)

**Keywords:** motor imagery, brain-machine interface, deep learning, obstacle detection

## Abstract

This study presents a human-computer interaction combined with a brain-machine interface (BMI) and obstacle detection system for remote control of a wheeled robot through movement imagery, providing a potential solution for individuals facing challenges with conventional vehicle operation. The primary focus of this work is the classification of surface EEG signals related to mental activity when envisioning movement and deep relaxation states. Additionally, this work presents a system for obstacle detection based on image processing. The implemented system constitutes a complementary part of the interface. The main contributions of this work include the proposal of a modified 10–20-electrode setup suitable for motor imagery classification, the design of two convolutional neural network (CNNs) models employed to classify signals acquired from sixteen EEG channels, and the implementation of an obstacle detection system based on computer vision integrated with a brain-machine interface. The models developed in this study achieved an accuracy of 83% in classifying EEG signals. The resulting classification outcomes were subsequently utilized to control the movement of a mobile robot. Experimental trials conducted on a designated test track demonstrated real-time control of the robot. The findings indicate the feasibility of integration of the obstacle detection system for collision avoidance with the classification of motor imagery for the purpose of brain-machine interface control of vehicles. The elaborated solution could help paralyzed patients to safely control a wheelchair through EEG and effectively prevent unintended vehicle movements.

## 1. Introduction

With recent advancements in artificial intelligence, an increasing number of studies have emerged focusing on the communication between computer systems and the human brain. In particular, research efforts have been directed toward the design and development of brain–computer interfaces (BCIs) and the accurate interpretation of motor imagery using electroencephalogram (EEG) signals. However, even with the utilization of deep learning models [1,2], these tasks pose significant challenges. Consequently, several studies have concentrated on enhancing the classification techniques employed in these domains. These included leveraging a one-dimensional convolutional neural network (CNN) [3,4], and more advanced architectural designs, such as multi-layer CNNs [5] or a deep residual CNN [6,7], which has exhibited significant success in these applications. Moreover, numerous research papers have investigated the integration of deep learning networks with steering interfaces, aiming to establish systems capable of translating users’ brain activity into movement instructions for vehicles, such as a hexapod [8], a telepresence robot control interface based on a support vector machine (SVM) [9,10], wheelchair control based on motor imagery and fuzzy logic [11], multi-scale CNNs [12], multilevel weighted feature fusion [13], and power spectrum estimation [14]. Despite the great progress in the field of interpreting human thoughts, the control over the vehicle is often limited to a single direction [8,15]. Despite the progress in decoding human brain activities, it remains challenging to implement BCIs in real-life applications.

The most practical use of BCIs in terms of vehicle control is to utilize brain activity to gain control over a wheelchair. Due to the nature of EEG signal processing, this task is very challenging. The main concerns include the latency of signal processing, a limited command set, user fatigue, and safety concerns. A delay between the user’s intention and the execution of a command in EEG-based control systems is very common. This latency can be problematic, especially in situations where quick responses are required, such as avoiding obstacles. EEG-based control systems often rely on a limited set of commands or actions that can be reliably detected from EEG signals. This limitation can restrict the range of tasks that can be performed. This may result in overcomplicated UIs for system control. However, the most vital condition to fulfill is the user’s safety. Errors or misinterpretations of EEG signals can lead to accidents or injuries, making it critical to implement robust fail-safe mechanisms. Despite these challenges, many researchers have tried to establish algorithms for wheelchair control. Many applications use P300 potential, as one of the most common and reliable ways of interpreting EEG signals.

The field of brain–computer interfaces (BCIs) has witnessed significant advancements in recent years, offering innovative solutions for addressing the challenges of controlling wheelchairs using brain signals. Several projects have explored the integration of BCIs with automated navigation techniques, to enhance the usability and reliability of brain-controlled intelligent wheelchairs. In [16], the author introduced a system that combines autonomous navigation with user-selectable destinations through motor imagery (MI) and P300-based BCIs, effectively reducing the mental burden on users and adapting to changing environments. Another work exploring event-related potentials tackled the issue of threshold-based EEG control by proposing a P300-based threshold-free brain switch, which demonstrated its efficacy for wheelchair control in both healthy individuals and patients with spinal cord injuries [17]. In their work, [18] Zhang et al. presented a similar idea of utilizing P300-based BCIs for destination selection and autonomous navigation, further alleviating the user mental burden and adapting to dynamic surroundings. Event-related potentials have proved to be reliable in terms of BCIs, but a more practical approach requires more direct communication, which can be provided by utilizing multi-modality. The combination of different modalities [19] like P300 potentials and SSVEP [20,21,22] or motor imagery [23,24,25] can increase the potential of BCIs. Exemplary applications can offer multiple control commands for both the direction and speed of wheelchairs [26]. These researches collectively demonstrated the potential of BCIs to revolutionize assistive technologies, making brain-controlled wheelchairs more accessible, reliable, and adaptable for individuals with diverse needs. However, they also revealed the great challenge of designing a reliable usable interface. Although modalities like P300 and SSVEP have demonstrated good accuracy, implementing them for steering a vehicle requires an additional screen-like interface. In this research, we decided to limit the EEG processing modalities to only movement imagery, to create a more natural interface for controlling a wheelchair. The approach presented in this paper aims to enhance the intuitiveness of the system and its reliability by combining a movement imagery BCI with an obstacle detection system for collision avoidance. The originality of this work is providing the potential user of a BCI controlled wheelchair with a safety mechanism that can avoid and/or take partial control over the wheelchair when the signal classification goes wrong and, as a result, puts the user into a potentially dangerous situation.The main contributions of this work include (1) the design of two convolutional neural network (CNN) models used for signal classification acquired from sixteen EEG channels; (2) the implementation of an obstacle detection system and its integration with a brain-machine interface; (3) demonstrating, probably for the first time, that visual transformers and depth estimation can significantly improve the control of a vehicle with thoughts.

## 2. Hardware Setup

**Figure 1:**
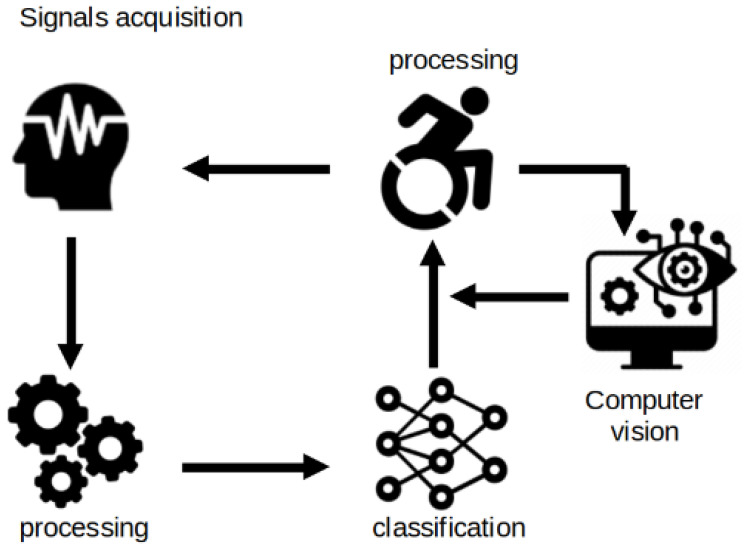
General overview of the system architecture.

The hardware utilized in this study plays an important role both during the signal acquisition phase as well as during testing a real-time application. A general overview of the system architecture is shown in Figure 1.

**Figure 2:**
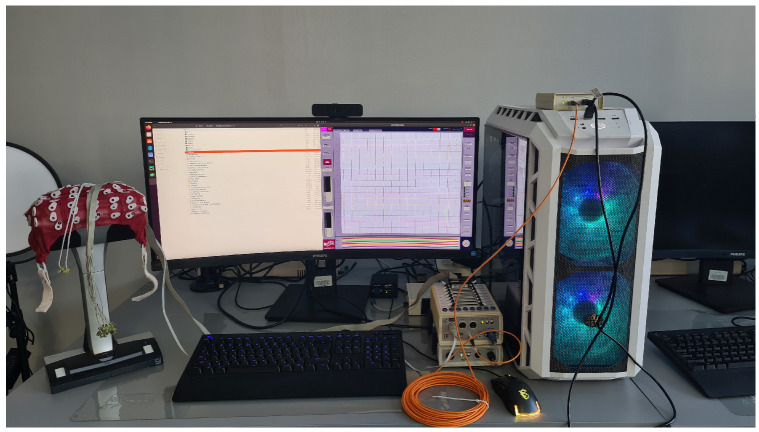
The Biosemi hardware and ActiView902 software utilized for EEG acquisition.

In this study, the electrical activity of the brain was recorded using a Biosemi ActiveTwo EEG system, BioSemi B.V., Amsterdam, The Netherlands and ActiView902 software (Figure 2). The sampling frequency employed was 2048 Hz, and the resolution of the EEG signals was 31.25 nV. Sixteen electrodes were utilized in conjunction with CMS (common mode sense) and DRL (driven right leg) electrodes. It should be underlined that the BioSemi utilizes a unique electrode configuration, replacing the conventional “ground” electrodes with two distinct electrodes. The suggested position of the CMS electrode is the center of the measuring electrodes, while the DRL should be placed away from the measuring electrodes. These two electrodes establish a feedback loop designed to drive the average potential of the subject (common mode voltage) as close as possible to the ADC reference voltage in the AD-box. The CMS/DRL loop offers additional functionalities that are challenging to achieve with a single standard ground electrode. Due to this feedback loop, the effective impedance of the DRL electrode is reduced by a factor of 100 at 50 Hz, resulting in a 40 dB extra common mode rejection ratio (CMRR) at 50 Hz compared to using normal ground electrodes with the same impedance. The DRL electrode serves as the sole current return path between the subject and the AD-box, with the return current electronically limited to 50 uA. This electronic limitation safeguards the subject from excessive currents resulting from amplifiers or electrode defects. Such an electrode configuration slightly differs from that typically used in similar experiments where the reference is an average between electrodes on the two ears.

The locations of the electrodes were determined using a modified International 10–20 system, with one electrode being relocated from the Oz position to the TP9 location. This altered electrode setup was a result of experiments aiming to determine which electrode exhibited the least differentiation in results between the right- and left-hand movement imagery.

To capture data during the acquisition sessions and facilitate real-time processing, a transmission control protocol (TCP) connection was established to route the data to a processing server. The TCP segment contained 128 samples, resulting in 16 packets being sent per second. A suitably prepared script was used to display the imagery movement activity commands (‘LEFT’, ‘RIGHT’, ‘RELAX’, ‘BREAK’). The vehicle for this project was built on top of an NVIDIA Jetson Nano computer, NVIDIA Santa Clara, CA, USA and a JetBot AI Kit Robot. The robot was equipped with an 8-megapixel wide-angle camera with 3280 × 2464 resolution.

**Figure 3:**
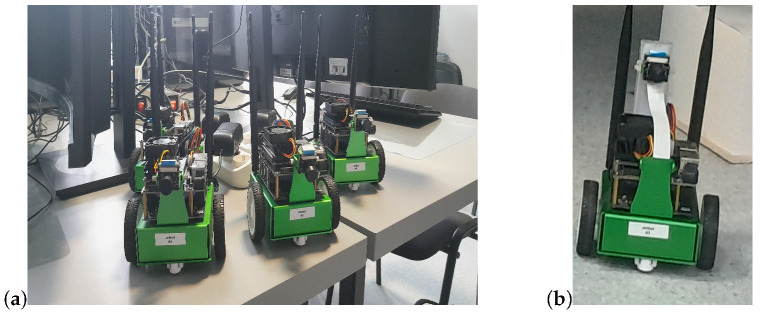
JetBot platforms prior to (**a**) and after modification (**b**).

The camera was moved from its original position to the back of the robot to improve the view of the robot’s surroundings and to increase the performance of the collision detection system. Both the JetBot and the server machine were connected to a local wireless network. During each test, the decisions generated by the server application were transmitted to the JetBot over a UDP protocol. The JetBot was programmed to move gradually every time it received corresponding information from the server (forward by a step of 5 cm and rotate left or right by an angle of 15 degrees). The original and modified JetBot platforms utilized in this study are presented in Figure 3.

## 3. Method

**Figure 4:**
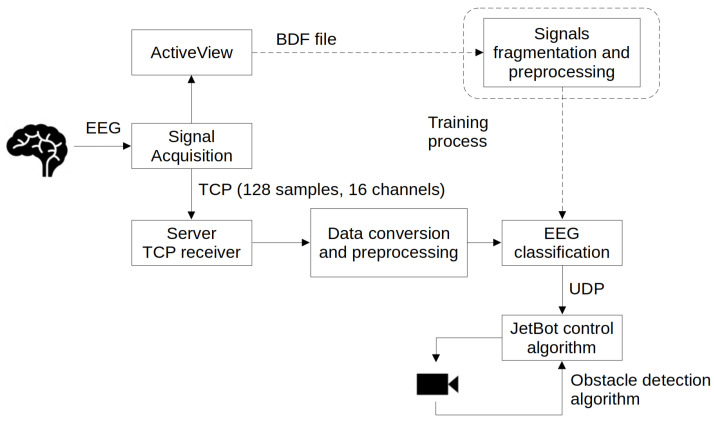
Conception diagram presenting the data flow and required protocols.

The general idea was to design a human-computer interface that elevates BCI-controlled vehicles to a level that allows reliable control. This required enhancing the BCI with an additional system. In general, the experiment relied on recording data with movement imagery, training models, implementing an obstacle detection system, and testing the interface in real time (with and without collision detection system support). A conceptual diagram representing the data flow, along with the required protocols and operations, is presented in Figure 4.

### 3.1. Data Acquisition

Data acquisition and experiments were carried out at the AI Living Lab, in the Department of Biomedical Engineering at Gdańsk University of Technology. The data acquisition process involved a series of 7 min trials. Three volunteers participated in the study: subjects S1, S2, and S3. Subjects S1 and S2 took part in two sessions, each consisting of ten trials, and one session with two trials. Subject S3 was recorded during a single session with two trials. The ten-trial sessions were used for model training, while the two-trial sessions served as testing data. Each trial consisted of 60 motor imagery tasks, evenly distributed across three classes, with randomized command orders. Each command (‘LEFT’, ‘RIGHT’, ‘RELAX’) lasted 4 s, followed by a 3 s break (‘BRAKE’). After each trial, subjects were given 3 min of rest. Sessions consisted of either 2 or 10 trials. During the motor imagery process, subjects were instructed to remain still. Limb movements were strictly prohibited. Subjects were allowed to imagine moving one of their arms in the manner most comfortable to them. The collected data comprised approximately 8 h of raw recordings, with 7 h dedicated to training data and 1 h for testing. During data acquisition sessions, ActiView902 software was used to store raw data in the BioSemi Data Format (BDF). Trial events were encoded using triggers in a dedicated channel, utilizing keyboard key presses, and simulated through proprietary software. ActiView902 provides the capability to transmit selected channels via a TCP socket. The volume of data sent in a single packet is contingent on the adjustable parameter setup in the ActiView902 software. Throughout the system testing and calibration phases, this parameter was set to 64 s. Setting the sampling rate to 2048 Hz and TCP segment to 128 samples resulted in the transmission of 16 packets per second. It is important to note that the ten-trial and two-trial sessions for the same subject were not recorded on the same day, ensuring variations in the electrode setup and environmental conditions. All the participating subjects agreed to take part in this research.

### 3.2. Data Preprocessing

The pre-processing pipeline was as follows: division into fragments, DC bias removal, unit conversion, filtering, downsampling, minimum value correction, logaritmization, and z-score normalization. The signal was referenced to 0.55×(C3 + C4), where C3 and C4 were the values read from the corresponding electrodes. This way of referencing the signal was adopted from the work [27].

**Figure 5:**
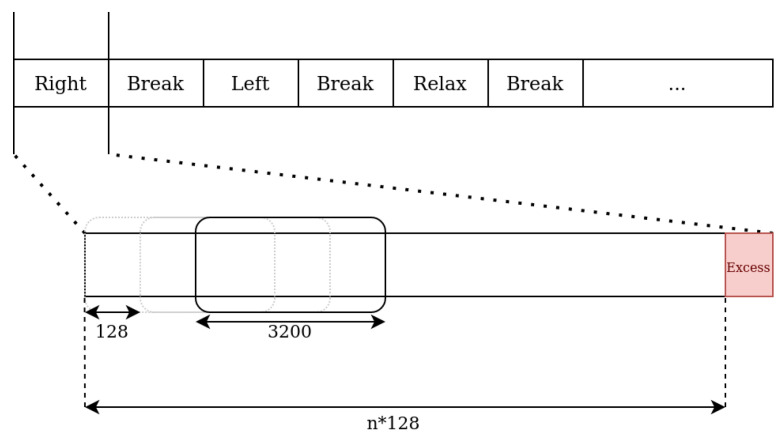
Visualization of the fragmentation process, where the numerical values represent the number of samples.

For the purpose of training the models, the recordings were divided into separate and non-overlapping fragments, based on the tags stored in the BDF file. Each fragment consisted of many samples divisible by 128, which corresponded to the number of samples transmitted in a single TCP segment. If a fragment did not meet this requirement, the excess samples at the end of the fragment were discarded. Next, a sliding window of size 3200 samples was moved along each fragment individually, with a step of 128 samples. The contents of the sliding window were then passed to the subsequent step in the pre-processing pipeline (Figure 5). This step was not performed on signals received during the real-time operation of the system. Instead, the last 3200 samples received were stored in a buffer, and further steps were carried out on its contents.

Elimination of the DC component was carried out by subtracting the average of the 8192 samples (4 s) preceding the last sample of the sliding window, in a given time step. DC bias removal was performed independently for each channel. Two digital Butterworth filters were used (a second-order low-pass filter with a cutoff frequency of 34.3 hertz, followed by a second-order high-pass filter with a cutoff frequency of 5.25 hertz). Signals from each channel were filtered and downsampled independently. The minimum value correction procedure in this study involved the following steps: Initially, the smallest value within a given time window was subtracted from the signal. Subsequently, the sample values were increased by a constant equal to $e^{-2}$. As a result, a signal with a minimum value equal to the mentioned constant was obtained. This correction procedure was applied individually to each channel.

### 3.3. Movement Imagery Classification Models

Two different networks were proposed. The first proposed architecture was a network performing the task of multi-class classification. The output of the network yielded assignment of the input signal fragment into one of three classes—right-hand movement intention, left-hand movement intention, and relaxation state.Models based on this architecture were trained on data containing only these three classes. The core of this network architecture was three convolution blocks: conv2D, avgPool2D, BatchNorm2D, Dropout2D layers. Next, there were Flatten, Linear, BatchNorm1d, Dropout, Linear, BatchNorm1d, Dropout layers. After the last layer of the network, the LogSoftmax activation function was applied. The negative log likelihood loss function was used.

During the real-time operation of the system that used a multi-class model at each timestep, which was every time a TCP segment containing 128 samples was received (every 62.5 ms), the model made a prediction based on the last 3200 samples stored in the EEG samples buffer. Every such prediction was stored in another buffer, which contained the 70 most recent predictions made by the model. The final decision of the EEG-based decision-making module was the prediction that appeared most frequently in the predictions buffer.

**Figure 6:**
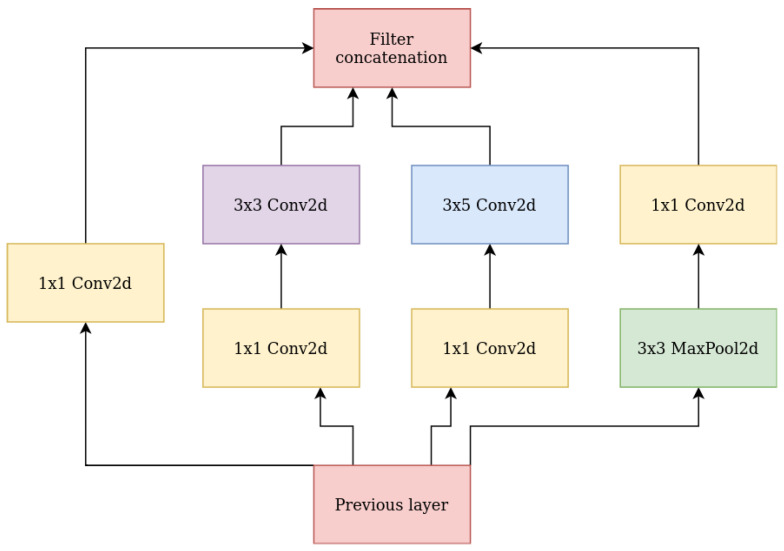
Modified inception module architecture.

The second tested architecture was a one vs. all network ensemble. A set of convolutional networks performed the task of two-class classification. Models based on this architecture detected one of the intentions, so it was necessary to train three models, one for each class. In addition to this, an auxiliary model was also trained, which detected signals that did not contain any of the intentions. The network architecture consisted of two convolution blocks of conv2D, avgPool2D, BatchNorm2D, and Dropout2D layers each. They were followed by four modified Inception [28] blocks, in which the 5 × 5 filter size was changed to 3 × 5. A schematic of the modified module is shown in Figure 6. After the last layer of the network, the sigmoid activation function was applied. The binary cross entropy loss function was used.

### 3.4. Obstacle Avoidance

**Figure 7:**
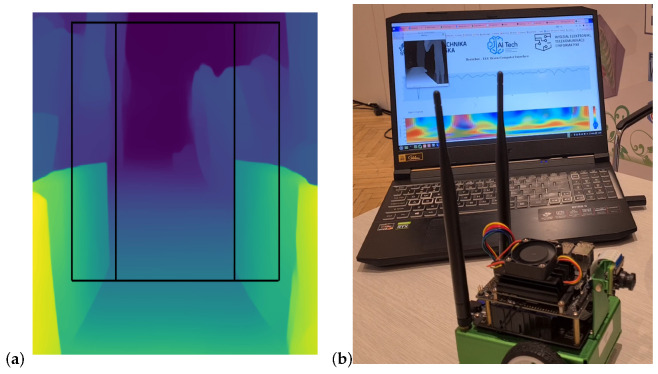
Exemplary result of Midas inverse depth estimation (**a**), software tests visualizing the movement imagery and detected obstacles (**b**).

An important element of our solution is the obstacle avoidance module, which enables the vehicle to safely navigate through its environment by identifying potential obstacles. The system utilized RGB images captured by the on-board camera, which were sent to the server and processed into an inverted distance map using Pytorch MiDaS v3.1 dpt_beit_large_512 model [29,30]. Due to the nature of the inverse depth estimation models, only the relative depth information was obtained from the inference. Inverse depth images were divided into three sections (Figure 7a): left, right, and center. The remaining parts of the image were ignored. The mean depth of each section was then calculated to determine if it exceeded a predefined threshold value. Cropping the bottom part of the sections proved beneficial for minimizing the chance of identifying floor segments as potential obstacles. The mean depth of each section was computed to determine if it surpassed the predefined threshold. This threshold was specifically set to facilitate the detection of large, encompassing objects such as walls or trash cans within the image. For identifying smaller obstacles, each section underwent subdivision into a square grid. The mean value of each square was calculated, and those values exceeding a specified threshold were tallied. The threshold value was set empirically during the robot calibration phase. Optimal values can vary slightly in different environments and should be fine-tuned for optimal performance.

$$P_{\mathrm{Left}}=\left\{\begin{array}{cc} 1 & \mathrm{if}\;D_{\mathrm{Left}}>T\\ 0 & \mathrm{otherwise} \end{array}\right.$$

(1) $$P_{\mathrm{Right}}=\left\{\begin{array}{cc} 1 & \mathrm{if}\;D_{\mathrm{Right}}>T\\ 0 & \mathrm{otherwise} \end{array}\right.$$

$$P_{\mathrm{Center}}=\left\{\begin{array}{cc} 1 & \mathrm{if}\;D_{\mathrm{Center}}>T\\ 0 & \mathrm{otherwise} \end{array}\right.$$

where $P_{Left},P_{Right},P_{Center}$ denote the probability of an object being present in the left, right, or center part of an image captured by the vehicle camera; and $D_{Left},D_{Right},D_{Center}$ denote the mean depth of the left, right, and center sections, respectively, while *T* is the predefined threshold for obstacle detection.

The obstacle detection information was combined with the subject motion intention to determine the final robot motion direction. When a vehicle is approaching an obstacle, it is necessary to decide if the driver’s intention should be overridden by the obstacle avoidance system. The general algorithm that was designed for this purpose is presented in Algorithm 1. In the conducted study, it was decided to use a binary probability of object presence estimated based on Equation (1).
**Algorithm 1** EEG overriding algorithm1:1. Initialize System:2:a. Start the EEG-based decision-making module.3:b. Start the obstacle avoidance module using computer vision.4:c. Set system parameters, including EEG accuracy threshold and obstacle detection thresholds.5:d. Read EEG signals and wait for the EEG-based decision-making modules buffers to fill.6:2. Loop:7:a. Read EEG signals and determine the user’s intended action (left, right, forward).8:b. Read RGB images from the onboard camera for obstacle detection.9:3. EEG Decision Processing:10:a. If EEG accuracy >= Probability of object presence:11:i. Proceed with the user’s intended action.12:b. Else:13:i. Pause the EEG-based decision-making temporarily.14:ii. Activate the obstacle avoidance module.15:4. Obstacle Avoidance:16:a. Process the RGB image to obtain an inverted distance map.17:b. Divide the map into left, right, and center sections.18:c. Calculate the mean depth of each section.19:d. If any mean depth exceeds the obstacle detection threshold:20:i. Override the user’s intended action with a “stop” command.21:ii. Implement obstacle avoidance maneuvers.22:5. User Feedback:23:a. Provide feedback to the user about the obstacle detection and avoidance.24:6. Resume EEG-Based Decision-Making:25:a. After a predefined time or obstacle-clearance condition, resume the EEG-based decision-making.26:b. Deactivate the obstacle avoidance module.27:7. End Loop.

If the driver expresses an intention to drive forward resulting in an unavoidable collision and an obstacle is detected in the middle box section, the obstacle avoidance module will react instantly by turning left or right to avoid the obstacle. The direction is determined based on a lack of obstacle detection in the left or right image sections. If an obstacle is detected in all boxes, the vehicle will turn right until the situation changes. The obstacle avoidance module is not activated when the user decides to go left or right.

## 4. Results

The proposed system contained several parts in need of evaluation. The deep learning models were trained and compared. The designed models were trained on several datasets containing samples recorded on different subjects. Both one-subject and multi-subject dataset combinations were tested. The accuracy and F1 score of the motor imagery classification for the proposed models are juxtaposed in Table 1.

To obtain a more comprehensive understanding of the classification models, recall and precision were also taken into consideration. The precisions and recalls calculated for particular folds are presented in Table 2.

It is worth mentioning that the very noisy data from the gaps between imaginary movements were also used to train the model, so that the models learned to label the falsity of this type of data.

The highest performance in terms of accuracy and F1 score was obtained for the multi-class model, which reached 83%.

In EEG-based BCIs, it is common that the system captures many relevant brain patterns but sometimes also tolerates some false positives. It is crucial to consider the practical implications of such scenarios. False positives might lead to unnecessary actions or commands, impacting the user experience. To limit or avoid such scenarios, we implemented an additional obstacle detection system whose function was to override the steering action in the case of such events. The results of a software test including visualization of both obstacle and movement imagery is presented in Figure 7b.

**Figure 8:**
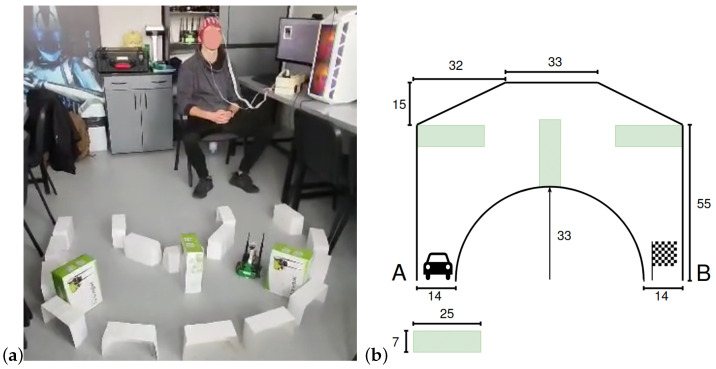
Exemplary test ride using EEG and the obstacle detection system (**a**), diagram of the racing track designed to test the interface (where: A is start, B is finish line, obstacles are marked green and all the measurements are in centimeters) (**b**).

An imperative part of the study was to test the system’s ability to steer the robot in a real-time scenario. The best-performing model was selected for the online tests. Therefore, the multi-class model trained on the S1 data was used. The obstacle detection system was used to improve the subject’s steering through disallowing straight movements into the detected obstacles. The training track shown in Figure 8 was constructed. The time for completing the track was measured.

Initially, five attempts at completing the route with the obstacle detection system turned ON and OFF were executed. The results of these trials can be seen in Table 3. With the obstacle detection system turned OFF, two attempts qualified as failed due to track limit violations (the operator went off the route or hit one of the obstacles), and the average time measured during the remaining three trials was 310 s.

With the obstacle detection system turned ON, the operator finished all five trials successfully, with an average time of 155.7 s. In conclusion, when the operator was not supported by the obstacle avoidance system, he struggled to travel through the track quickly and safely.

The object detection was evaluated through a series of experiments (50 per object) with a diverse set of objects, including natural objects (plants) and artificial objects (bottles, boxes, elements of walls). All tests were performed using the Midas small 3.0 model. Two distinct lighting scenarios were considered in these experiments: good lighting and poor lighting. Good lighting was defined as daylight conditions where the camera image is not noisy and objects in the image are clearly visible. Poor lighting was defined as illumination conditions where objects visible in the image are slightly noisy and the details of the objects are less visible.

It is worth mentioning that the obstacle detection system, which used images from an RGB camera, repeatedly prevented hitting an obstacle during the trials. Overall, the implemented system detected and prevented hitting an obstacle 24 times during the trial sessions. This indicated that the use of obstacle detection in the solution for controlling the vehicle using EEG signals gave satisfactory results.

To obtain further understanding of how the BCI could be utilized for controlling a vehicle or wheelchair, we conducted a comparison between various methods and their integration with a driving support system. We focused on comparing the number of electrodes and the method utilized for acquiring information from EEG signals, as well as the accuracy, vehicle control type, and integration with the obstacle detection system. The results of this analysis are presented in Table 4.

## 5. Discussion

In this study, we introduced a novel framework aimed at enhancing the wheelchair-driving experience for patients through the utilization of movement imagery. Our primary objectives revolved around the precise detection of user intention through analyzing EEG signals from a limited number of electrodes, as well as the enhancement of the safety aspects of BCI-controlled vehicles or interfaces. One of the most common methods for detecting intention based on movement imagery is the common spatial pattern algorithm and its modifications. This, however, requires at least an 18-electrode setup [33]. Our emphasis, however, lay in minimizing the electrode count. To achieve this, we systematically explored various alternative methods. We showcased the efficacy of artificial neural networks in discerning movement imagery and translating it into driving intentions. To accommodate the constraints of our designed interface’s 16-channel EEG setup, we tailored the complexity of our neural network models accordingly. Among the two proposed approaches, the multi-class model exhibited the highest accuracy. It is noteworthy that our training dataset incorporated a fusion of signals from diverse subjects and a strategic approach to model robustness. The desired scenario involves training a model on a generalized dataset and subsequently fine-tuning it for specific users. However, this approach proves challenging, due to the substantial variations in signals obtained from different users. Our investigations consistently reflected this challenge, with each inclusion of data from diverse users leading to a decline in overall accuracy. Notably, a significantly higher accuracy was achieved with a subject-dependent approach, though this necessitated individual designing and training of the system for each user. We evaluated our solution on a relatively small group of users. It is important to acknowledge the inherent challenges associated with utilizing BCI technology. The effectiveness of a BCI is contingent upon factors such as the user’s cognitive capabilities, attention, and the ability to voluntarily modulate brain activity. Extensive training is required for users to acquire proficiency in controlling a BCI system using their brain signals.

Despite the limited size of our participant group, we conducted comprehensive evaluations of classification models across various folds, assessing accuracy, F1-score (see Table 1), recall, and precision (see Table 2). Notably, in the majority of the tested scenarios, the achieved recall surpassed the precision, suggesting a tendency of the tested models to prioritize sensitivity over precision. This implied that the models excel in identifying most actual positive cases but may exhibit a tendency to include some false positives, thereby lowering the precision. Such occurrences are not uncommon and underscore the significance of implementing algorithms, such as the one proposed in our study, capable of mitigating false positive classifications in EEG-based BCI applications. Furthermore, we conducted a comparative analysis between our proposed solution and existing EEG-based BCI applications designed for wheelchair or vehicle control. The findings, as illustrated in Table 4, demonstrated the challenges associated with achieving high accuracy in movement imagery classification when using a limited number of electrodes. Notably, while the solution outlined in [8] exhibited slightly higher accuracy compared to ours, it is essential to emphasize that our approach accommodates a greater number of distinct classes. This comparison underscores a key advantage of our method, as it obviates the need for an additional user interface for selecting the desired direction or location for wheelchair navigation, seamlessly integrating EEG classification into the control process.

It should be mentioned that all signals were recorded in controlled laboratory conditions. The lab’s location rendered recording sessions susceptible to electromagnetic interference. Additionally, varying times of day during the subsequent recording sessions may have influenced brain activity. While evaluating the precise impact of these factors is challenging, their variability potentially contributed to the improved generalization capabilities of our model. It needs to be underlined that we established the efficacy of movement imagery for steering using surface EEG signals. This technique has been widely endorsed, particularly in events like Cybathlon BCI racing [31,32], where participants must independently generate and control multiple control commands for computer-based racing scenarios. Despite advancements in BCI technology, its reliability remains insufficient for patients to control real vehicles, even in controlled competitions like Cybathlon. Accurate classification of user intention might not be sufficient for designing interfaces that allow control over real vehicles in a real-life environment. Our innovation lies in the integration of BCI with a driving support system based on computer vision. To illustrate the benefits of this combination, we engaged users in steering a small robot instead of a full-size wheelchair—a safer alternative for both the driver and the environment. This approach afforded greater flexibility in designing test routes. Our study demonstrated that the combination of a BCI with a video-based obstacle avoidance system enabled users to navigate the test route faster and without collisions. We demonstrated the applicability of the solution using the JetBot platform. It must be underlined that transitioning from a small robot (JetBot) to a full-size wheelchair in real-world applications will involve important safety and ethical considerations. Ensuring the well-being of users and bystanders is paramount. Before implementing the technology in a larger context, a rigorous safety assessment and risk analysis should be conducted. This includes evaluating the reliability of the BCI system, obstacle avoidance algorithms, and the overall responsiveness of the BCI-controlled wheelchair.

The application of our trained models over the course of several weeks demonstrated a robust performance, with no notable decline. Nonetheless, the process of designing and testing a model for EEG-based movement imagery classification introduces noteworthy challenges, especially concerning its prolonged applicability. Difficulties may arise over extended periods, influenced by factors such as user fatigue, diverse mental states, and fluctuating environmental conditions. Addressing these challenges requires a thorough examination of the model’s resilience to prolonged usage, considering the dynamic nature of EEG signals and their susceptibility to external factors.

In our study, the decision to focus exclusively on obstacle avoidance, rather than exploring scenarios involving approaching and stopping next to obstacles, was driven by the need for a targeted and streamlined investigation. This choice allowed us to delve deeply into the effectiveness of the combined BCI and obstacle avoidance system for facilitating smooth navigation from point “A” to “B”, ensuring a comprehensive and in-depth analysis of the core functionalities without introducing unnecessary complexity or variables.

## 6. Conclusions

We successfully developed a brain-machine interface that enables vehicle control through movement imagery. Our approach, which involved tailoring the models to each individual subject, yielded the best results. However, it is important to note that the usability of the model varied among subjects. The achieved accuracy of 83% is comparable to state-of-the-art solutions in the field. The integration of a collision detection system, utilizing movement imagery and a 16-channel EEG, proved to be a valuable addition to conventional robot control. We demonstrated that the operator was able to go through the test track twice as fast when controlling the vehicle through movement imagery with obstacle detection system support. While acknowledging that the presented solution will need modifications and further studies prior to its implementation in a full-size wheelchair, our findings demonstrate its potential for effectively preventing unintended vehicle movements. This underscores the applicability of our solution in real-world scenarios, emphasizing the need for continued refinement and comprehensive investigations for seamless integration into larger-scale applications.

## Figures and Tables

**Table 1:** Model performance comparison—accuracy and F1 score.

		Multi-Class Model		One vs. All Model	
**Train Fold**	**Test Fold**	**Accuracy**	**F1 Score**	**Accuracy**	**F1 Score**
S1	S1	0.83	0.83	0.81	0.81
S2	S2	0.72	0.72	0.81	0.81
S1 + S2	S1	0.80	0.80	0.74	0.76
S1 + S2	S2	0.71	0.66	0.70	0.70
S1 + S2	S3	0.66	0.66	0.64	0.61

**Table 2:** Models performance comparison—precision and recall.

		Multi-Class Model		One vs. All Model	
**Train Fold**	**Test Fold**	**Precision**	**Recall**	**Precision**	**Recall**
S1	S1	0.82	0.84	0.78	0.84
S2	S2	0.72	0.72	0.80	0.82
S1 + S2	S1	0.78	0.82	0.74	0.78
S1 + S2	S2	0.66	0.66	0.66	0.74
S1 + S2	S3	0.66	0.66	0.57	0.65

**Table 3:** Experimental results of traversing the test route with the obstacle detection system turned OFF and ON.

	obstacle detection sys. OFF				
Test number	1	2	3	4	5
Time [s]	failed	failed	270	360	300
	obstacle detection sys. ON				
Test number	1	2	3	4	5
Time [s]	287.8	127.7	122.2	120.0	120.9

**Table 4:** Comparison of BCIs for wheelchair/vehicle control.

Method	Number of Electrodes	Electrode System	Number of Tested Subjects	Accuracy [%]	EEG Device	Information about Vehicle Control	Obstacle Detection or Avoidance System	Reference
movement imagery MSPCSP	32	10–20	18	85	Enobio-32, NE Neuroelectrics, Barcelona, Spain	Direct/single direction	no	[15]
movement imagery CNN-LSTM	16	10–20	4	86	Emotiv EPOC+, Emotiv, San Francisco, CA, USA	Direct/single direction	no	[8]
movement imagery FBCSP	32	10–20	1	above 90	g.Nautilus research, g.tec GmbH, Schiedlberg Austria	Direct	no	[31]
movement imagery CSP	16	10–10	2	52 (66 for healthy subject)	g.LADY bird, g.tec GmbH, Schiedlberg Austria	Direct	no	[32]
P300 and motor imagery	15	10–20	5	77	NuAmps, Compudemics Neuroscan, Victoria, Australia	Direct	no	[26]
P300 and motor imagery	15	10–20	6(P300)/3(MI)	-	NuAmps, Compudemics Neuroscan, Victoria, Australia	User selects a destination on the screen by means of appropriate UI	Yes	[16]
P300	32	10–20	5	94	NuAmps, Compudemics Neuroscan, Victoria, Australia	User selects a destination on the screen by means of appropriate UI	Yes	[17,18]
motor imagery CNN	16	10–20	4	83	Biosemi ActiveTwo EEG system, BioSemi B.V., Amsterdam, The Netherlands	Direct	Yes	our method

## Data Availability

The code and data utilized in this study are available on reasonable and qualified research request. Every inquiry will undergo an assessment. Upon acceptance, signing an access agreement will be required.
